# What the papers say

**DOI:** 10.1093/jhps/hnag049

**Published:** 2026-10-10

**Authors:** Ali Bajwa

**Affiliations:** 30 New Road, Impington, Cambridge, CB24 9EL, United Kingdom

## Abstract

The Journal of Hip Preservation Surgery (JHPS) is not the only place where work in the field of hip preservation can be published. Although our aim is to offer the best of the best, we are continually fascinated by work, which finds its way into journals other than our own. There is much to learn from it, and so JHPS has selected six recent and topical subjects for those who seek a summary of what is taking place in our ever-fascinating world of hip preservation. What you see here are the mildly edited abstracts of the original articles, to give them what JHPS hopes is a more readable feel. If you are pushed for time, what follows should take you no more than 10 min to read. So here goes …

## Morphometric and cellular evaluation of delaminated cartilage from the acetabulum

The authors from Germany [1] note that delaminated acetabular cartilage flaps are associated with inferior outcomes of arthroscopic treatment of femoroacetabular impingement syndrome (FAIS). Recently, concomitant arthroscopic minced cartilage implantation has shown encouraging early clinical results under such circumstances.

The purpose of their study was to investigate the morphology, outgrowth, and functionality of chondrocytes from delaminated acetabular cartilage flaps in comparison to controls, in order to assess their suitability for minced cartilage implantation. It was hypothesized that despite morphologic differences, chondrocyte viability and functionality do not differ between groups.

Delaminated acetabular chondral flaps were obtained from 11 patients undergoing hip arthroscopy. Control samples were taken either from the acetabulum in patients undergoing total hip arthroplasty for avascular necrosis (2 samples) or from patients undergoing minced cartilage implantation at the knee (12 samples). Synchrotron radiation microcomputed tomography analyses and histologic evaluations were conducted. *In vitro* investigations assessed cell proliferation, metabolic activity, and redifferentiation potential in three-dimensional spheroid cultures.

Microcomputed tomography revealed a significantly increased mean number of chondrocyte lacunae in delaminated acetabular cartilage compared to control samples, along with a significant reduction of lacunar diameter. Morphometric and histologic analyses demonstrate cell-free, necrotic areas with fiber structures within delaminated acetabular cartilage samples. Regarding the number of outgrown cells, metabolic activity, and proliferation capacity, no differences between delaminated acetabular cartilage and control samples were observed. Initially cultured chondrogenic spheroids of cells from delaminated acetabular cartilage samples had lower protein and proteoglycan content compared to controls, but no differences were observed for spheroids cultured with Transforming Growth Factor Beat (TGF-β1) supplementation.

The authors concluded that delaminated acetabular cartilage exhibits altered morphology. However, cell numbers, outgrowth potential, and population doublings of chondrocytes isolated from delaminated acetabular cartilage were comparable to those of controls. Redifferentiation of chondrocytes isolated from delaminated acetabular cartilage relies on TGF-β1 stimulation. Delaminated acetabular flaps are strongly degenerated, characterized by atrophic chondrocyte lacunae and tissue necrosis, indicating that autograft utilization is unfavorable. However, cell outgrowth and functionality suggest that delaminated acetabular cartilage flaps provide a suitable tissue source for minced cartilage implantation after augmentation with TGF-β1.

## Labral hypoplasia by preoperative magnetic resonance imaging predicts higher revision and arthroplasty risk after hip arthroscopy for femoroacetabular

In this study, the authors from New York, USA [2] aim to determine the association between labral width as measured on preoperative magnetic resonance imaging (MRI) and patient-reported outcomes, achievement of clinically significant thresholds, and reoperation rates in hip arthroscopy for FAIS at minimum 10-year follow-up.

A retrospective review of a prospectively gathered database of hip arthroscopy patients from August 2012 to June 2014 was conducted. Inclusion criteria were patients ≥18 years with clinically and radiographically confirmed FAIS and labral tearing who underwent primary hip arthroscopy with labral repair or debridement and had ≥10 years of follow-up. MRI labral width measurements were performed by 2 blinded musculoskeletal radiologists at standardized clockface locations using a validated technique. Outcomes were assessed using the modified Harris Hip Score (mHHS) and Non-Arthritic Hip Score (NAHS). Patients were classified as hypoplastic if they had a labral width below the mean on two or more views. Outcomes and reoperation rates were compared between groups using independent samples t-tests for continuous variables and chi-square tests for categorical variables.

A total of 83 patients (83 hips; 65.9% follow-up) with a mean age of 42.93 years and body mass index of 25.22 kg/m^2^ were included, with a mean follow-up of 11.3 years. Patients were categorized into hypoplastic (*n* = 42) and nonhypoplastic (*n* = 41) groups. There was no significant difference between hypoplastic and nonhypoplastic groups with respect to age, sex, smoking status, or intraoperative procedures. Additionally, there were no significant intergroup differences in mHHS or NAHS improvement at 5 or 10 years postoperatively. Both groups showed high achievement of the mHHS minimal clinically important difference threshold at 10-year follow-up with no significant difference (nonhypoplastic: 90.3% vs hypoplastic: 85.2%). There was also no difference achievement of the patient acceptable symptom state (nonhypoplastic: 64.5% vs. hypoplastic: 70.4%). However, the hypoplastic group had a significantly higher rate of revision arthroscopy (28.6% vs 9.8%) and conversion to total hip arthroplasty (21.4% vs 4.9%) when compared with the nonhypoplastic group. The authors thus concluded that the hypoplastic labral width on preoperative MRI was associated with an increased risk of revision hip arthroscopy and conversion to total hip arthroplasty at 10-year follow-up in patients with FAIS.

## Association of central acetabular osteophytes with microinstability and increased combined anteversion in borderline dysplasia hips

The authors from China and Japan [3] state that central acetabular osteophytes (CAOs) have been implicated in abnormal hip biomechanics. However, their relationship with microinstability in borderline developmental dysplasia of the hip (BDDH) is unclear. The purpose of this case–control study was to compare radiographic parameters between BDDH hips with and without CAOs and to determine whether CAOs are associated with microinstability. It was hypothesized that CAOs would correlate with increased version abnormalities and microinstability.

They retrospectively reviewed patients with BDDH, defined as a lateral center-edge angle of 18° to 25°, who underwent hip arthroscopy from 2020 to 2024. Patients were grouped as BDDH with CAO (cBDDH) or without CAO (nBDDH). Radiographic parameters, including anterior/posterior acetabular coverage, femoral neck version, acetabular version, and combined anteversion, were compared. Intraoperative findings of microinstability and ligamentum teres (LT) pathology were analysed. Binary logistic regression identified predictors of CAO.

A total of 157 patients were included (cBDDH, 76; nBDDH, 81). The cBDDH group demonstrated higher rates of microinstability and LT tears, as well as increased femoral neck, acetabular, and combined anteversion. Logistic regression showed that microinstability (β = .027), LT tear (β = .010), and combined anteversion (β = 1.343; *R*^2^ = 0.542) were associated with CAO. They concluded that in BDDH, CAOs were strongly associated with microinstability, LT tears, and increased combined anteversion. CAO may serve as a radiographic marker of instability and early osteoarthritis in BDDHs.

## Biomechanical comparison of native acetabular labrum, fresh-frozen meniscus, and fresh-frozen anterior tibialis tendon for labral reconstruction

The authors from Missouri, USA [4] note that the acetabular labral tears can lead to hip pain and dysfunction and progress to osteoarthritis if left untreated. While surgical repair is the gold standard, reconstruction with grafts is necessary in cases of irreparable labral damage. Clinical data suggest that meniscal allografts may offer superior outcomes as compared with anterior tibialis tendon allografts, but biomechanical comparisons remain limited.

Their controlled laboratory study aimed to compare the tissue properties of the native acetabular labrum, fresh-frozen medial meniscal allograft, and fresh-frozen anterior tibialis tendon allograft. The null hypothesis was that the fresh-frozen medial meniscus will not have statistically different biomechanical properties to the native labrum and fresh-frozen anterior tibialis tendon.

Samples of the native labrum (*n* = 6), fresh-frozen meniscus (*n* = 6), and fresh-frozen anterior tibialis tendon (*n* = 6) were standardized and biomechanically tested using a 3-phase protocol on an Instron materials testing machine. Properties measured included ultimate strength, yield force, yield strength, stiffness, Young modulus, and work to failure. One-way analysis of variance with post hoc Tukey was performed to determine statistically significant differences among tissue types.

Ultimate strength, yield force, and Young modulus were not significantly different across tissue types. Native labrum exhibited significantly higher yield strength (mean ± SD; 48.25 ± 14.56 MPa) when compared with the meniscus (26.69 ± 6.51 MPa) and anterior tibialis tendon (27.50 ± 5.02 MPa; *P* = .002). Stiffness was greater in the anterior tibialis tendon (159.35 ± 44.11 N/mm) than in the native labrum (89.07 ± 26.76 N/mm) and meniscus (112.84 ± 56.74 N/mm). Work to failure was highest in the native labrum (1143.62 ± 551.31 N·mm) *versus* the meniscus (310.44 ± 168.29 N·mm) and tibialis tendon (265.93 ± 131.48 N·mm).

The authors concluded that the stiffness of the medial meniscus tested closer to the native labrum than the anterior tibialis, but both grafts had significantly lower yield strength and work to failure than the native labrum. Together, the results indicate that the medial meniscus and anterior tibialis are viable graft options, but they do not replicate the tissue properties of the native labrum.

## Ligamentum teres tear with hyaline body formation: a rare cause of mechanical hip pain

The authors from Aleppo, Syria [5] note that the traumatic injuries of the LT are an under recognized cause of hip pain and instability. Diagnosis is often delayed due to nonspecific symptoms and normal radiographs. Complete LT rupture associated with intra-articular hyaline body formation is rare and may result in chronic mechanical symptoms. They report a case of a 38-year-old female who presented with progressive right hip pain and limping over 2 years following direct blunt trauma. Symptoms evolved into mechanical pain, instability, and restricted motion despite conservative management. Clinical examination revealed an antalgic gait, positive Trendelenburg sign, and reduced hip rotation. Radiographs were unremarkable, while MRI suggested LT injury with joint effusion. Open surgical exploration *via* a modified Gibson approach revealed a complete LT rupture with multiple intra-articular hyaline bodies within the acetabular fossa, contributing to femoral head instability. Surgical management included excision of loose bodies, reduction, and capsular stabilization.

The authors state that the case highlights a rare post-traumatic combination of complete LT rupture and hyaline body formation leading to chronic instability. It emphasizes the importance of maintaining clinical suspicion in patients with persistent mechanical symptoms despite normal imaging. In resource-limited settings, open surgical approaches provide effective diagnosis and treatment when arthroscopy is unavailable. They concluded that Post-traumatic LT rupture with hyaline bodies is a rare but treatable cause of chronic hip instability. Early recognition and appropriate surgical management can lead to excellent functional outcomes.

## Increased risk of proximal femur fracture following hip arthroscopy with increased age and tobacco use

The authors from Los Angeles, USA [6] aim to determine the incidence and risk factors for proximal femur fracture following hip arthroscopy. Using the PearlDiver Mariner 170 Ortho database, patients undergoing hip arthroscopy between 2010 and 2023 were identified *via* Current Procedural Terminology codes. Subsequent proximal femur fractures at time points 30 days, 90 days, 6 months, and 1 year postoperatively were identified with International Classification of Diseases, Ninth and Tenth Revision codes. Demographic factors and comorbidities were compared between patients with and without fractures. Univariate and multivariate logistic regression analyses were performed.

Among 100 397 hip arthroscopy patients, 994 (0.99%) sustained a proximal femur fracture. Patients with fractures were significantly older (mean age 49 vs 39.5 years) and had higher rates of diabetes (22% vs 8%), tobacco use (41% vs 19%), and obesity (29% vs 17%). On multivariate analysis, independent risk factors included tobacco use (adjusted odds ratio, 2.95; 95% confidence interval, 1.87–4.65) and increasing age (adjusted odds ratio per year, 1.045; 95% confidence interval, 1.039–1.051). The authors concluded that proximal femur fractures occur in approximately 1% of hip arthroscopy patients. Older age and tobacco use independently increase fracture risk.

## References

[1] Gebhardt S, Schoon J, Bortel E et al. Morphometric and cellular evaluation of delaminated cartilage from the acetabulum. Orthop J Sports Med 2026;14:23259671261450542. 10.1177/23259671261450542 PMC13389100 42488192

[2] Berzolla E, Chen L, Messina J et al. Labral hypoplasia by preoperative magnetic resonance imaging predicts higher revision and arthroplasty risk after hip arthroscopy for femoroacetabular impingement syndrome at 10 year follow-up. Arthroscopy 2026. 10.1002/arj.70404 [Online ahead of print] 42391555

[3] Zhu J-B, Ding R, Huang Y et al. Association of central acetabular osteophytes with microinstability and increased combined anteversion in borderline dysplasia hips. Am J Sports Med 2026;54:2527–34. 10.1177/03635465261453074 42343539

[4] Garlapaty AR, Lehenbauer C, Bezold W et al. Biomechanical comparison of native acetabular labrum, fresh-frozen meniscus, and fresh-frozen anterior tibialis tendon for labral reconstruction. Orthop J Sports Med 2026;14:23259671261434226. 10.1177/23259671261434226 PMC13219856 42221204

[5] Kortibi M, Razzouk Q, Issa M et al. Ligamentum teres tear with hyaline body formation: a rare cause of mechanical hip pain. Trauma Case Rep 2026;64:101365. 10.1016/j.tcr.2026.101365 PMC13217891 42221137

[6] Lian J, Zafar-Khan A, Banffy MB. Increased risk of proximal femur fracture following hip arthroscopy with increased age and tobacco use. Arthroscopy 2026;42:1664–8. 10.1002/arj.70317 42219868

